# Carvacrol Improves Vascular Function in Hypertensive Animals by Modulating Endothelial Progenitor Cells

**DOI:** 10.3390/nu15133032

**Published:** 2023-07-04

**Authors:** Tays Amanda Felisberto Gonçalves, Viviane Silva Lima, Arthur José Pontes Oliveira de Almeida, Alinne Villar de Arruda, Ana Caroline Meneses Ferreira Veras, Thaís Trajano Lima, Evyllen Myllena Cardoso Soares, Adhonias Correia dos Santos, Maria Eduarda Costa de Vasconcelos, Mathania Silva de Almeida Feitosa, Robson Cavalcante Veras, Isac Almeida de Medeiros

**Affiliations:** Department of Pharmaceutical Sciences, Federal University of Paraiba, João Pessoa 58059-900, PB, Brazil; taysamanda@ltf.ufpb.br (T.A.F.G.); vivianelima@ltf.ufpb.br (V.S.L.); arthur-jp@hotmail.com (A.J.P.O.d.A.); alinnevillar@ltf.ufpb.br (A.V.d.A.); carolmeneses@ltf.ufpb.br (A.C.M.F.V.); thais.trajano@academico.ufpb.br (T.T.L.); evyllen.cardoso@academico.ufpb.br (E.M.C.S.); adhonias.correia@academico.ufpb.br (A.C.d.S.); mecv@academico.ufpb.br (M.E.C.d.V.); mathaniarez@ltf.ufpb.br (M.S.d.A.F.); robsonveras@ccs.ufpb.br (R.C.V.)

**Keywords:** oxidative stress, endothelial progenitor cells, endothelial dysfunction

## Abstract

Carvacrol, a phenolic monoterpene, has diverse biological activities, highlighting its antioxidant and antihypertensive capacity. However, there is little evidence demonstrating its influence on vascular regeneration. Therefore, we evaluated the modulation of carvacrol on endothelial repair induced by endothelial progenitor cells (EPC) in hypertension. Twelve-week-old spontaneously hypertensive rats (SHR) were treated with a vehicle, carvacrol (50 or 100 mg/kg/day), or resveratrol (10 mg/kg/day) orally for four weeks. Wistar Kyoto (WKY) rats were used as the normotensive controls. Their systolic blood pressure (SBP) was measured weekly through the tail cuff. The EPCs were isolated from the bone marrow and peripherical circulation and were quantified by flow cytometry. The functionality of the EPC was evaluated after cultivation through the quantification of colony-forming units (CFU), evaluation of eNOS, intracellular detection of reactive oxygen species (ROS), and evaluation of senescence. The superior mesenteric artery was isolated to evaluate the quantification of ROS, CD34, and CD31. Treatment with carvacrol induced EPC migration, increased CFU formation and eNOS expression and activity, and reduced ROS and senescence. In addition, carvacrol reduced vascular ROS and increased CD31 and CD34 expression. This study showed that treatment with carvacrol improved the functionality of EPC, contributing to the reduction of endothelial dysfunction.

## 1. Introduction

Endothelial progenitor cells (EPC), precursors of mature endothelial cells, are capable of exerting functional effects on arteries, capillaries, and veins, which include maintenance of the endothelial layer and vascular homeostasis, as well as participation in vascular regeneration and neovascularization [1].

Under normal conditions, most EPC remains in the bone marrow, relatively inactivated, in contact with stromal cells. However, in response to an injury through chemotactic factors, EPCs are released from the bone marrow and mobilized to the injured tissue directly into the damaged vessel structures, preventing blood vessel dysfunction [2,3]. In contrast, when the percentage of EPC decreases, or its function is impaired, the angiogenic capacity is weakened, reducing its regeneration capacity and potentiating vascular injury [4].

A series of experimental and clinical studies have been conducted in the context of hypertension that demonstrate reductions in functionality and proliferative capacity [5] with lower numbers of circulating EPCs [6,7,8]. The pathological mechanisms involving EPC dysfunction in arterial hypertension are complex and include abnormalities of the oxidant and antioxidant system, which leads to the increased production of reactive oxygen species [9]. A fundamental point of this process is that prolonged exposure to high levels of ROS induces EPC dysfunction, causing premature cell senescence driven by telomere wear, making the regeneration process difficult [10]. Thus, attenuating oxidative stress by antioxidant molecules may maintain the functional integrity of EPC [11].

There is an increasing search for products of natural origin that act as the biological aspect, helping the individual’s well-being in several areas of science. In this sense, evidence has linked the Mediterranean diet with a variety of benefits to cardiovascular health [12]. Origanum vulgare (oregano), an herb commonly used in this diet, has been shown to possess various antioxidant, anti-inflammatory, antidiabetic, and antinociceptive activities [13]. Such activities can be mainly attributed to the components present in this herb’s essential oil [14]. Carvacrol, the main constituent of the essential oil of oregano, stands out for its various pharmacological activities, such as an antioxidant properties [15], including on the cardiovascular system [16,17,18].

Carvacrol leads to reduced levels of inflammatory factors [19,20], inhibits the formation of atheroma plaques [21], increases the levels of antioxidant enzymes and activation of endothelial nitric oxide synthase (eNOS) [22], and reduces senescence cell [23]. Furthermore, carvacrol instigates the angiogenesis of human mesenchymal cells by modulating the differentiation of these cells [24].

Therefore, the main objective of this work was to evaluate the regenerative potential of carvacrol through EPC modulation in spontaneously hypertensive rats.

## 2. Materials and Methods

### 2.1. Animal and Experimental Design

Male Wistar Kyoto (WKY) and Spontaneously Hypertensive Rats (SHR), 12 weeks old, were housed in temperature-controlled conditions (21 ± 1 °C), with a 12-h light–dark cycle and with free access to food (NUVILAB CR-1, QUIMTIA^®^, Curitiba, PR, BR) and water. The animals came from the Animal Production Unit of IPerFarm/UFPB and the Central Animal Facility/UFAL. The Committee on Ethics and Use of Animals (CEUA) from the Federal University of Paraíba under no. 2171120320 approved the current experimental protocols.

WKY animals were used as a negative control (WKY-CTL; *n* = 8). As a model of hypertension, we used SHR animals, which have changes in EPC functionality associated with impaired vascular function [25]. The SHR animals were randomly divided into four experimental groups: hypertensive control (SHR-CTL; *n* = 8), hypertensive treated with carvacrol 50 mg/kg (SHR-C50; *n* = 8) or 100 mg/kg (SHR-C100; *n* = 8), and hypertensive treated with resveratrol 10 mg/kg (SHR-RE10; *n* = 8). All experimental groups received daily doses of the respective treatments for four weeks.

### 2.2. Indirect Measurement of Systolic Blood Pressure (SBP) and Heart Rate (HR)

The tail plethysmography method was used to detect the SBP and HR at the start and end of each treatment [26]. Before the measurements, the rats were allowed a 10-min period to acclimate to the heated (28–30 °C) acrylic box environment. After this period, the animals were transferred to an acrylic container, and the tail cuff and piezoelectric sensor were connected to the tail for pulse measurements.

The piezoelectric sensor signals were amplified using ADinstruments^®^ (Bella Vista, NSW, Australia) and digitized using suitable software (Labchart7^®^). In addition, the SBP and HR values were calculated using the average of three consecutive measurements.

### 2.3. Isolation and In Vitro Culture of EPC Derived from Bone Marrow and Peripheral Blood

After the animal was anesthetized, 10 mL of peripheral blood (PB) with EDTA was collected in the hepatic portal vein to obtain circulating mononuclear cells [27,28]. After euthanasia, the femur and tibia were separated and dissected [29]. Subsequently, the epiphyses were removed, and the bone cavities were washed with 10 mL of PBS/EDTA. Then, the cells were centrifuged at 600× *g* for 10 min at 4 °C. Next, the supernatant was discarded, and the pellet was resuspended in 10 mL of medium 199 (Sigma-Aldrich^®^, St. Louis, MO, USA). Finally, the blood was collected, and bone marrow (BM) washing was added to the Ficoll^®^-Paque PLUS density gradient media (Cytiva Life Sciences, Uppsala, Sweden) for cell separation. Thus, the mononuclear cell layer was used for flow cytometry to quantify, characterize, and follow seven-day cell cultures.

Suspensions with at least 95% viability were transferred to 6-well plates, in the proportion of 1 × 10^6^ mononuclear cells/1 cm^2^/0.5 mL of EBM-2 supplemented with endothelial growth factors such as human epidermal growth factor (hEGF), vascular endothelial growth factor (VEGF), basic human fibroblast growth factor (hFGF-b), insulin-like growth factor-1 (IGF-1), hydrocortisone, gentamicin and amphotericin, 2% fetal bovine serum, ascorbic acid, and heparin (Lonza, Walkersville, MD, USA) and kept in a 5% CO_2_ incubator at 37 °C for 24 h. In addition, nonadherent cells were plated in 24-well plates, previously prepared with fibronectin (1 μg/mL), in proportions of 1 × 10^6^ cells/1 cm^2^/0.5 mL of EBM-2. Every 2 days, the culture medium was changed until the seventh day, whereas the experimental protocols were followed.

### 2.4. EPC Quantification and Characterization after Treatment with Carvacrol

After isolating cells from the peripheral blood and bone marrow, cells were diluted to reach a ratio of 10^6^/100 μL. Then, the cells were labeled with the following antibodies: FITC-anti-VEGFR-2 (1:50; Santa Cruz Biotechnology, Dallas, TX, USA), plus PE-anti-CD34 (1:50; Santa Cruz Biotechnology), plus APC-anti-CD133 (1:10; BioLegend, Inc., San Diego, CA, USA), and PerPCP-Cy5-7-AAD (0.25 µg/106; Cayman Chemical, Ann Arbor, MI, USA). After adding the antibodies, the cells were incubated for 30 min and the 7-AAD for 10 min at 4 °C away from light. Each analysis was performed on the population of 100,000 events using a FACS Canto-II cytometer (BD, Santa Monica, CA, USA).

### 2.5. Functional Evaluation of Endothelial Progenitor Cells (EPCs) by Colony-Forming Units

Following carvacrol treatment, the EPC clonogenicity properties were evaluated. Thus, to determine the number of colony-forming units (CFU), the cells were visually inspected after seven days through an inverted microscope with 40× magnification (NIKON Eclipse TS100, Tokyo, Japan). The results were expressed as a percentage of the average number of colonies formed by 10^6^ plated cells. Two different observers manually calculated the average number of colonies from ten wells under a light microscope.

### 2.6. Evaluation of EPC-eNOS Expression after Carvacrol Treatment

The expression of total endothelial nitric oxide synthase (eNOS) and phosphorylated endothelial nitric oxide synthase (ps1177) are performed by the flow cytometry technique as previously described [30]. In brief, after seven days of cell culture, EPC was fixed with 4% paraformaldehyde for 20 min at 37 °C, following permeabilization with 0.1% Triton X-100 for 10 min at 37 °C. Then, a blocking solution consisting of PBS and 5% BSA was added for 2 h at room temperature. Subsequently, the EPCs were resuspended and incubated with primary anti-eNOS (1:50; Bd Biosciences, Franklin Lakes, NJ, USA) or anti-eNOS ps1177 (1:100; Bd Biosciences, Franklin Lakes, NJ, USA) antibodies for 1 h at room temperature. Finally, the samples were washed with PBS and incubated with PE-conjugated anti-mouse IgG (Invitrogen™, Waltham, MA, USA) for 1 h in the dark. The samples were analyzed using a FACS Canto-II cytometer (BD, Santa Monica, CA, USA).

### 2.7. Evaluation of the Effect of Carvacrol Treatment on ROS Production

Superoxide anions (ROS) were measured by a dihydroethidium (DHE) probe (Sigma-Aldrich, St. Louis, MO, USA). DHE, an oxidative fluorescent dye, was oxidized to ethidium bromide and intercalated into DNA in the presence of superoxide anions. Briefly, cultured EPC or superior mesenteric artery sections (10 μm) from different groups were loaded with 10 μM DHE for 40 min at 37 °C in the dark. Subsequently, the samples were washed with PBS and analyzed by fluorescence microscopy (NIKON Eclipse TS100, Tokyo, Japan). The analysis of ROS production in EPC was performed by two different analyzers, quantifying the fluorescence intensity using NIS elemental^®^ software version 4.2 through the observations of 10 other fields. Data were normalized by the control group.

### 2.8. Evaluation of Cellular Senescence by Flow Cytometry

To measure SA-β-gal activity by flow cytometry, the fluorogenic substrate C12FDG (5-dodecanoylaminofluorescein Di-β-D-galactopyranoside, Invitrogen, Life Technologies SAS; Waltham, MA, USA) was used as previously described [31]. Briefly, the EPC was pretreated for 1 h with chloroquine (300 μM) to raise the pH of the lysosomes (pH = 6.0), plus an additional 1 h of C12FDG (33 μM) in the dark. Finally, EPCs were washed with ice-cold PBS, trypsinized, and immediately analyzed with a FACS Canto-II (BD, Santa Monica, CA, USA).

### 2.9. Immunofluorescence

Initially, superior mesenteric artery segments of the treated animals were included in Tissue Tek Compound (OCT), frozen in liquid nitrogen, and kept at −80 °C until the experimental protocols. Subsequently, superior mesenteric artery sections (10 μm) from different groups were fixed with 4% paraformaldehyde for 30 min at 37 °C. Next, the vessels were blocked with PBS with 5% BSA for 30 min at room temperature. Finally, monoclonal antibodies were incubated: FITC-conjugated anti-PECAM antibody (1:500; Santa Cruz Biotechnology, Dallas, TX, USA) and PE-conjugated anti-CD34 antibody (1:500; Santa Cruz Biotechnology, Dallas, TX, USA) overnight at 4 °C. Subsequently, the sections were washed with PBS and mounted with DAPI mounting medium (Fluoroshield™, Sigma Aldrich, St. Loius, MO, USA) for nuclear identification [32]. Data were acquired using fluorescence microscopy (NIKON Eclipse TS100, Tokyo, Japan) and analyzed with ImageJ^®^ software version 1.50i.

### 2.10. Statistical Analysis

All data were expressed as the mean plus or minus the standard error of the mean (e.p.m). The significant difference between the tested groups was tested by one-way or two-way analysis of variance (ANOVA), followed by Tukey’s post-test. Data were considered significant when *p* < 0.05. All analyses performed were calculated using the statistical program Graph Pad Prism version 7.0^®^.

## 3. Results

### 3.1. Carvacrol Reduces SBP in SHR Animals

Changes in the SBP of WKY and SHR animals from the different experimental groups after four weeks of treatment are shown in Table 1. At the beginning of the treatment, the baseline SBP of the WKY-CTL group was significantly lower than the SHR-CTL (Table 1), SHR-C50, SHR C100, and SHR-RE10 groups (*p* > 0.05). The pressure levels of the WKY-CTL group remained lower than the SHR-CTL group (*p* > 0.05) at the end of the treatment. The SHR-C50, SHR-C100, and SHR-RE10 groups significantly reduced SBP compared to the SHR-CTL group at the end of the treatment (*p* < 0.05). Similarly, the SHR-C50, SHR-C100, and SHR-RE10 groups significantly reduced SBP compared to the respective groups at the beginning of the treatment. Thus, carvacrol effectively reduced SBP in hypertensive animals.

### 3.2. Carvacrol Enhances EPC Mobilization to the Peripheral Circulation

**Figure 1:**
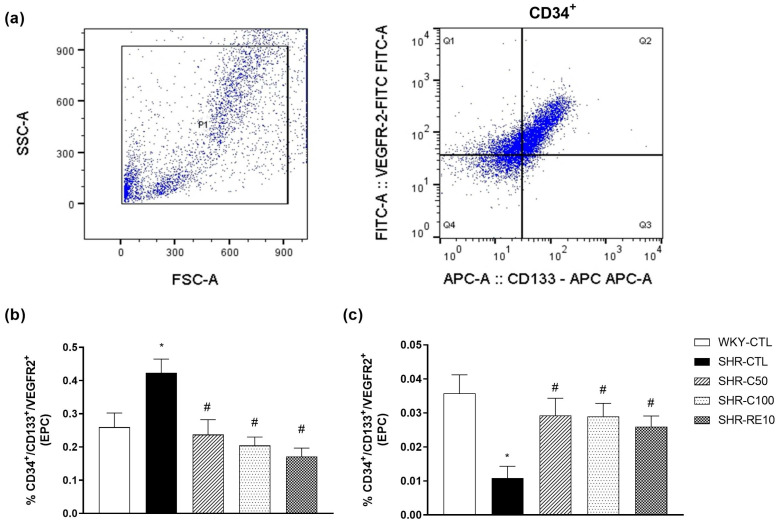
Quantification and characterization of EPC after treatment with carvacrol. Representative images of EPC characterization by flow cytometry in the bone marrow (**a**). Quantification of EPC isolated from bone marrow (**b**) and peripheral circulation (**c**). EPC levels were expressed as the percentage of CD34^+^/CD133^+^/VEGFR2^+^ cells in relation to mononuclear cells. * *p* < 0.05 vs. WKY-CTL; ^#^ *p* < 0.05 vs. SHR-CTL. Results are expressed as the mean ± e.p.m. The ANOVA test was used for the statistical analysis, followed by Tukey’s post-test.

EPC was quantified in the bone marrow and peripheral circulation by expressing surface antigens CD34^+^/CD133^+^/VEGFR-2^+^ (Figure 1a). Hematopoietic cells were selected based on the forward and side scatter values, excluding debris, aggregates, and dead cells [33]. The EPC levels in the bone marrow were lower in the WKY-CTL group (0.26 ± 0.04, *n* = 4) compared to the hypertensive SHR-CTL group (0.42 ± 0.04, *n* = 4) (*p* < 0.05) (Figure 1b). Treatments with carvacrol at doses of 50 mg/kg (0.23 ± 0.04, *n* = 6) and 100 mg/kg (0.20 ± 0.03, *n* = 5) were similar to that of the WKY-CTL group and significantly different from the SHR-CTL group (*p* < 0.05). The SHR-RE10 group (0.17 ± 0.03, *n* = 5) was also significantly different from the SHR-CTL group (*p* < 0.05) (Figure 1b).

On the other hand, the circulating EPC levels were lower in the SHR-CTL group (0.011 ± 0.004, *n* = 5) compared to the normotensive WKY-CTL group (0.036 ± 0.005, *n* = 4) (*p* < 0.05) (Figure 1c). Interestingly, treatments with carvacrol at doses of 50 mg/kg (0.029 ± 0.005, *n* = 4) and 100 mg/kg (0.029 ± 0.005, *n* = 5) significantly improved EPC circulation compared to the SHR group -CTL (*p* < 0.05). Similarly, the SHR-RE10 group (0.026 ± 0.003, *n* = 4) also showed an increase in the circulating number of EPCs (Figure 1c). These results indicated that carvacrol reduced the entrapment of EPC in the bone marrow, favoring its migration to the peripheral circulation.

### 3.3. Characterization of EPC after Cell Culture

**Figure 2:**
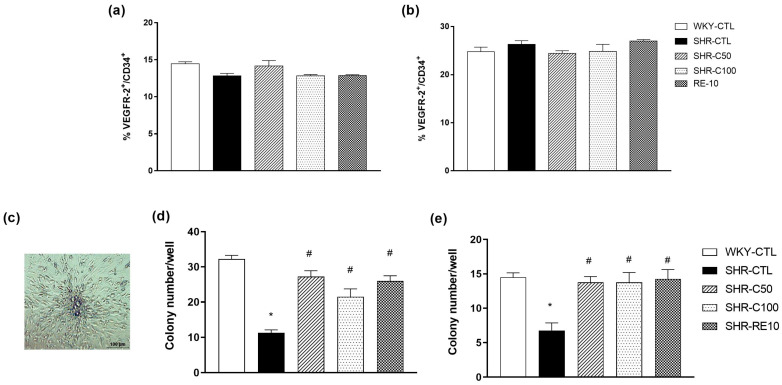
Effect of carvacrol treatment on CFU expression in EPC cultured 7 days after treatment. Characterization of the EPC population confirmed by the expression of VEGFR-2 and CD34 in the bone marrow, (**a**) peripheral circulation, and (**b**) cultured cells. Representative image of CFU evaluated after seven days of cell culture (**c**). Number of CFU after seven days of cell culture of cells isolated from bone marrow (**d**) and peripheral circulation (**e**). Results are expressed as the mean ± e.p.m. The ANOVA test was used for the statistical analysis, followed by Tukey’s post-test. * *p* < 0.05 vs. WKY-CTL; ^#^ *p* < 0.05 vs. SHR-CTL.

After 7 days of cell culture, EPC was characterized in the bone marrow and peripheral circulation. The results obtained after evaluating the expression of the CD34 and VEGFR-2 surface antigens demonstrated the achievement of EPC in all the groups under study, with no statistically significant differences between the treated groups (Figure 2a,b).

Treatment with carvacrol increased the proliferative capacity of EPC derived from the bone marrow and peripheral circulation. After seven days of cultivation, the results indicated that the SHR-CTL group (BM = 11.2 ± 0.8, *n* = 4; PB = 6.7 ± 1.1, *n* = 4) (Figure 2d,e) markedly reduced the colony-forming units (CFU) when compared to the WKY-CTL group (BM = 32.2 ± 1.0, *n* = 4; PB = 14.5 ± 0.6, *n* = 4) (*p* < 0.05). Interestingly, this effect was reversed by the treatments of the SHR-C50 group (BM = 27.2 ± 1.6, *n* = 4; PB = 13.7 ± 0.8, *n* = 4), SHR-C100 (BM = 21.5 ± 2.2, *n* = 4; PB = 13.7 ± 1.4, *n* = 4), and SHR-RE10 (BM = 26.0 ± 1.4, *n* = 4; PB = 14.2 ± 1.3, *n* = 4). Therefore, these results indicate that carvacrol improved the clonogenicity of EPC.

### 3.4. Carvacrol Treatment Increases eNOS Activity and Expression

**Figure 3:**
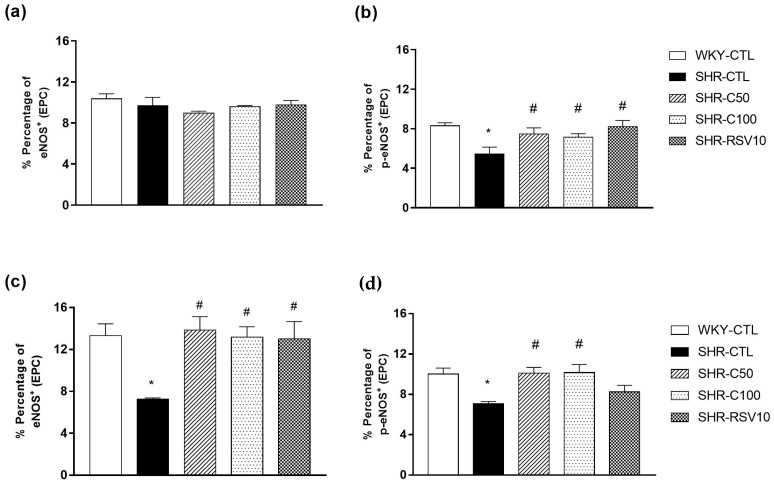
Evaluation of the total eNOS and phosphorylated eNOS in EPC cultured 7 days after treatment. Evaluation of the total eNOS (**a**) and p-eNOS (**b**) expression in cultured EPC from bone marrow. Quantification of the total eNOS (**c**) and p-eNOS (**d**) expression in EPC isolated from the peripheral circulation. Results are expressed as the mean ± e.p.m. The ANOVA test was used for the statistical analysis, followed by Tukey’s post-test. * *p* < 0.05 vs. WKY-CTL; ^#^ *p* < 0.05 vs. SHR-CTL.

The depletion in NO levels is linked to the low repair capacity mediated by EPC. In contrast, NO signaling mediates the induction of proliferation, angiogenesis, and migration of EPC to the peripheral circulation. Thus, we evaluated the expression and activity of eNOS, the enzyme that synthesizes NO. In EPC isolated from bone marrow, the different experimental groups did not show significant changes in eNOS expression (*p* > 0.005) (Figure 3a). In contrast, the percentage of phosphorylated eNOS (p-eNOS) was significantly reduced in the SHR-CTL group (BM = 5.50 ± 0.6; *n* = 4) compared to the WKY-CTL group (BM = 8.34 ± 0.2; *n* = 5) (Figure 3b). Treatments with carvacrol at doses of 50 mg/kg (BM = 7.50 ± 0.5; *n* = 4) and 100 mg/kg (BM = 7.17 ± 0.3; *n* = 4) were able to reverse the impairment of eNOS activity characterized in the hypertensive group. Treatment with resveratrol (BM = 8.42 ± 0.5; *n* = 5) was similar to the groups treated with carvacrol.

In the EPC isolated from the peripheral circulation, we verified a reduction in the percentage of total eNOS and p-eNOS in the SHR-CTL animals (eNOS = 7.2 ± 0.1%, *n* = 6; p-eNOS = 7.0 ± 0.2%, *n* = 6) when compared to the WKY group -CTL (eNOS = 13.3 ± 1.1%, *n* = 6; p-eNOS = 10.0 ± 0.5%, *n* = 6) (Figure 3c,d). In contrast, the SHR-C50 (eNOS = 13.8 ± 1.2%, *n* = 5; p-eNOS = 10.1 ± 0.5, *n* = 6), SHR-C100 (eNOS = 13.1 ± 0.9%, *n* = 5; p-eNOS = 10.1 ± 0.8, *n* = 5), and SHR-RE10 groups (eNOS = 13.0 ± 1.6%, *n* = 5; p-eNOS = 8.42 ± 0.6, *n* = 5) (Figure 3c,d) significantly increased in eNOS expression and activity, showing similar levels to the WKY-CTL group. The results revealed that enhanced EPC functionality may be related to increased eNOS activation.

### 3.5. Carvacrol Reduced Oxidative Stress and Cellular Senescence in EPCs

Elevated ROS levels, such as oxidative stress conditions, lead to reduced cell migration due to, at least in part, an increase in senescence and cell apoptosis, in addition to reducing the mobilization, migration, and adhesion factors. Therefore, we evaluated the fluorescence intensity by quantifying superoxide anions in EPC after 7 days of cultivation using the DHE probe.

**Figure 4:**
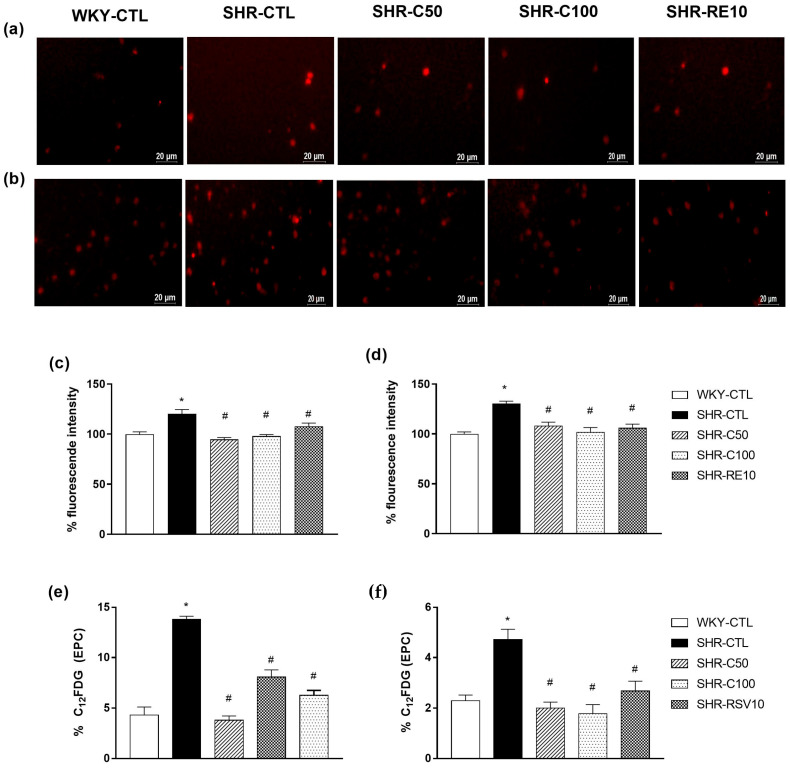
Effect of carvacrol treatment on ROS production and cellular senescence in different experimental groups in EPC cultured 7 days after treatment. Representative images of DHE fluorescence in EPC from MO (**a**) and SP (**b**). Quantification of fluorescence intensity of EPC isolated from MO (**c**) and SP (**d**) after seven days of cultivation. Evaluation of cellular senescence by quantifying the fluorescence intensity of C12FDG from EPC isolated from MO (**e**) and SP (**f**). Results are expressed as the mean ± e.p.m. The ANOVA test was used for the statistical analysis, followed by Tukey’s post-test. * *p* < 0.05 vs. WKY-CTL; ^#^ *p* < 0.05 vs. SHR-CTL.

The DHE probe emitted basal fluorescence in EPC-cultured cells in the different experimental groups. In the SHR-CTL group, an increase in basal fluorescence intensity (120.1 ± 4.3%; *n* =6) was verified when compared to the WKY-CTL group (100.0 ± 2.3%; *n* = 6) (Figure 4a,c) in bone marrow-derived EPC. Interestingly, the SHR-C50 (94.7 ± 1.8%; *n* = 6), SHR-C100 (98.1 ± 1.4%; *n* = 6), and SHR-RE10 (107.7 ± 3.2%, *n* = 4) groups showed fluorescence similar to the WKY-CTL group.

Similar results were observed in the EPC derived from the peripheral circulation, where the levels of DHE fluorescence intensity of the WKY-CTL group (100.0 ± 2.1%; *n* = 6) showed no significant difference between the SHR-C50 (108.1 ± 3.5%; *n* = 6), SHR-C100 (102.0 ± 4.4%; *n* = 6), and SHR-RE10 groups (106.1 ± 3.7%, *n* = 6) (Figure 4b,d). However, the SHR-CTL group (130.5 ± 2.3%; *n* = 6) showed an increase in the basal production of superoxide anions compared to the other experimental groups.

Cellular senescence decreases the functionality and migration of EPC, hampering its ability to restore endothelial dysfunction. Thus, we evaluated senescence through the activity of SA-β-gal through the C12FDG probe. As a result, the SHR-C50 (BM = 3.8 ± 0.4, *n* = 6; PB = 2.0 ± 0.2, *n* = 6) and SHR-C100 (BM = 6.2 ± 0.4, *n* = 5; PB = 1.7 ± 0.3, *n* = 6) groups and SHR-RE10 (BM = 8.1 ± 0.5, *n* = 4; PB = 2.6 ± 0.3, *n* = 6) prevented increase induced by the SHR-CTL group (BM = 13.8 ± 0.3, *n* = 4; PB = 4.7 ± 0.4, *n* = 6) during SA-β-gal activity (Figure 4e,f), showing levels similar to the WKY-CTL group (BM = 4.3 ± 0.5, *n* = 4; PB = 2.3 ± 0.2, *n* = 6) both in bone marrow-derived EPC and in peripheral circulation EPC. Considering these results, we can infer that carvacrol negatively modulated cellular senescence due to reduced oxidative stress.

### 3.6. Carvacrol Reduces Vascular Oxidative Stress and Induces EPC-Mediated Reendothelialization

**Figure 5:**
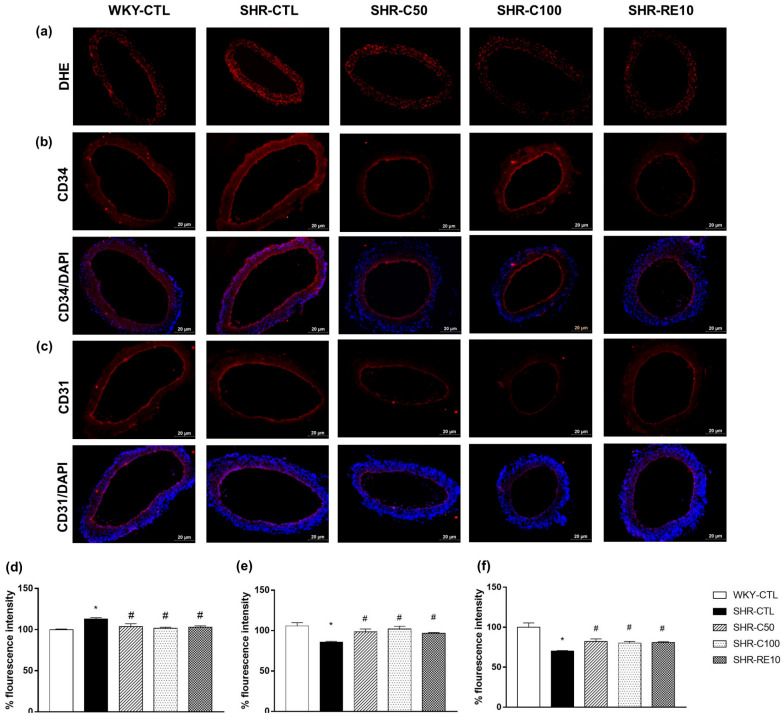
Assessment of vascular oxidative stress and reendothelialization after the treatment. Representative images of the fluorescence intensity of DHE (**a**), CD34 (**b**), and CD31 (**c**) in superior mesenteric arteries isolated from the treated animals. The cell nucleus (blue) was stained with DAPI. Bar graph expressing the measure of the relative intensity of the basal fluorescence (%) of DHE (**d**), CD34 (**e**), and CD31 (**f**) in histological sections of the superior mesenteric artery. Results are expressed as the mean ± E.P.M. Data were analyzed using the one-way ANOVA statistical test, followed by Tukey’s post-test. * *p* < 0.05 vs. WKY; ^#^ *p* < 0.05 vs. SHR.

Vascular oxidative stress is related to a lower recovery of the endothelial layer, as it can damage adjacent endothelial cells and reduce the cell adhesion capacity. In our study, the SHR-CTL animals (112.9 ± 1.5; *n* = 6) showed an increase in the intensity of DHE fluorescence compared to the WKY-CTL group (100 ± 0.6; *n* = 6) (Figure 5a,d). Interestingly, the treatments of the SHR-C50 (101.9 ± 1.9; *n* = 6), SHR-C100, and SHR-RE10 groups were able to reduce the fluorescence intensity observed in the SHR-CTL group (*p* < 0.05). These results show that carvacrol inhibited the production of ROS characteristic of hypertension, reducing vascular damage.

The improvement in EPC functionality was directly related to the increase in regenerative capacity. Thus, we evaluated the expressions of CD34 and CD31 in the blood vessels of treated animals. SHR-CTL animals showed a reduction in CD34 (Figure 5b,e) and CD31 (Figure 5c,f) fluorescence intensity compared to WKY-CTL animals. However, this effect was reversed by treating animals SHR-C50, SHR-C100, and SHR-RE10. Therefore, these results indicate that carvacrol improved endothelial dysfunction in the treated hypertensive animals.

## 4. Discussion

A critical component of the Mediterranean diet includes the herb oregano, which is commonly added to salads, spaghetti, soups, and sauces and is also used in the preparation of meats, sausages, and canned foods and to preserve cucumbers, mushrooms, and tomatoes [12]. Essential oils are responsible for the main pharmacological properties attributed to oregano, with carvacrol as the main constituent [34]. This monoterpene has been extensively studied and has been shown to present several benefits, including antioxidant [35], anti-inflammatory [36], and cardioprotective [17,22,37] properties, especially antihypertensive action [18,38], as demonstrated by our results.

Furthermore, in the present study, carvacrol prevents endothelial dysfunction in hypertensive animals by upregulating EPC mobilization, increasing the proliferative and functional capacity, and reducing the oxidative stress and senescence of cells isolated from bone marrow and peripheral blood, which contribute to EPC-mediated reendothelialization.

Multiple data indicate that EPC has diverse surface markers, which depend on the EPC maturation levels. Furthermore, EPC is a heterogeneous group of cells characterized by expressing on their surface CD133 and CD34 as hematopoietic progenitor cell markers and VEGFR2 as an endothelial lineage marker, characterizing the most reliable cell marking [39]. Thus, to characterize the EPC population, we adopted the concomitant labeling of CD133^+^, CD34^+^, and VEGRF-2^+^.

When quantifying the number of EPCs, we verified that carvacrol reduced the entrapment of EPCs in the bone marrow, making them more available to perform endothelial repair in the peripheral circulation. Studies have reported that increased EPC mobilization in the circulation aims to preserve the endothelial integrity in response to vascular damage [8]. The increase in EPC migration is associated with functional maintenance, a necessary condition for their repair capacity [40]. Thus, carvacrol efficiently increased the mobilization of EPC to the peripheral circulation, improving cell function. Similar results were observed by treatment with resveratrol, as demonstrated by Huang, who reported an increase in the biological process of EPC [41].

The clonogenic capacity of EPC has been used as an index of functional analyses, being correlated as a cardiovascular risk factor when it presents a low formation of CFU [9]. In hypertension, the decline in the functionality of the EPC occurs primarily in the reduction of circulating EPC, and this factor is accentuated as hypertension progresses [42]. The low functionality of these cells has been reported in patients with hypertension that is difficult to control and with damage to the target organs [43,44,45]. In our study, we also demonstrated that hypertensive animals have a deficiency in repair mediated by these cells by reducing the proliferation of these cells, corroborating with studies in SHR animals [46,47] and hypertensive patients [48,49,50].

Furthermore, treatment with carvacrol increased the proliferative capacity of EPC similarly to animals treated with resveratrol and the normotensive group. Studies have demonstrated that consuming resveratrol can enhance the proliferative capacity of EPCs through increasing nitric oxide (NO) signaling [41].

NO is an essential mediator for angiogenesis by inducing EPC mobilization and improving the migratory and proliferative capacity [4]. This fact was evidenced through experiments with knockout eNOS-/- mice, which failed in the mobilization of EPC to the peripheral circulation [51]. The malfunction in NO bioavailability is a characteristic of endothelial dysfunction, which depends on the balance between eNOS production and inactivation triggered by ROS. Interestingly, carvacrol treatment not only repaired the inactivation of the PI3K/AKT-eNOS-NO pathway but also attenuated ROS production in SHR EPCs. The NO-mediated pathway can promote the growth and mobilization of EPC from the bone marrow into the peripheral circulation [52,53]. Thus, we suggest that carvacrol increases the EPC migratory capacity by reversing the damage to the NO signaling pathway in hypertensive animals.

Experimental studies have indicated that hypertensive patients have high rates of ROS formation associated with increased cellular senescence [54,55]. Thus, when produced in the bone marrow, due to the deficiency of chemotactic factors and the accelerated state of senescence, the mobilization of these cells does not occur properly, becoming trapped in the bone marrow and intensifying the endothelial dysfunction [56]. Therefore, we demonstrated that carvacrol reduced cellular senescence, causing an increase in the migratory capacity of EPC and making them available to perform the repair of damaged organs.

The antioxidant capacity of carvacrol [20] acts beneficially on the cardiovascular system, restoring vascular function [18,38] and reducing the activity of the NADPH oxidase enzyme, the main enzyme involved in the formation of ROS [21]. Recently, carvacrol has been shown to increase SOD activity in human mesenchymal stem cells [24]. Thus, we can suggest that the increase in performance observed in EPC isolated from hypertensive animals treated with carvacrol is associated with its antioxidant capacity.

Marketou et al. reported that EPC plays an important role in arterial stiffness and remodeling in hypertensive patients, where reduced mobilization reflects inefficient repair [57]. Therefore, we investigated how the increase in EPC mobilization induced by carvacrol altered vascular remodeling. In SHR animals, treatment with carvacrol reversed vascular oxidative stress, which may contribute to the increase in EPC-mediated reendothelialization, as demonstrated by the increase in CD31- and CD34-labeled cells in the vessels of treated hypertensive animals. Similar results were observed with SHR-RE10, corroborating previous studies demonstrating that resveratrol accelerates the reendothelialization of balloon-injured arterial segments [58]. 

Furthermore, Matluobi et al. showed through in vitro experiments that carvacrol increased the migration, angiogenesis, and transdifferentiation of mesenchymal cells to endothelial cells. This effect was mediated by an increase in the expression of VEGF, a critical chemotactic factor involved in EPC-mediated reendothelialization [24]. However, in our study, the expression of chemotactic factors was not quantified, which is the main limitation. Therefore, additional studies are needed to prove this hypothesis and better elucidate the mechanisms by which carvacrol modulates EPC function and the reendothelization process.

## 5. Conclusions

This study elucidated the effects of carvacrol on EPC-mediated endothelial repair, improving mobilization, proliferation, and cell function; increasing eNOS expression; and reducing oxidative stress and cell senescence. However, additional studies to evaluate the intracellular signaling mechanisms involved in the regenerative potential of carvacrol are necessary to consolidate its protective action in the cardiovascular system.

## Figures and Tables

**Table 1:** SBP (mmHg) measurements at the beginning and the end of the treatments for the different experimental groups.

Groups	Initial SBP	Final SBP
WKY-CTL (*n* = 6)	138 ± 2.8	137 ± 3.4
SHR-CTL (*n* = 7)	186 ± 5.1 *	202 ± 5.1
SHR-C50 (*n* = 6)	193 ± 3.7 *	171 ± 3.8 ^# † ‡^
SHR-C100 (*n* = 7)	188 ± 3.2 *	163 ± 2.7 ^# † ‡^
SHR-RE10 (*n* = 6)	194 ± 3.2 *	168 ± 5.2 ^# † ‡^

Results are expressed as the mean ± e.p.m. The ANOVA test was used for the statistical analysis, followed by Tukey’s post-test. * *p* < 0.05 vs. initial WKY-CTL; ^#^ *p* < 0.05 vs. final WKY-CTL; ^†^ *p* < 0.05 vs. final SHR-CTL; ^‡^ *p* < 0.05 vs. start of treatment.

## Data Availability

The data presented in this study are available on request from the corresponding authors.
